# Herbicide-Resistant Invasive Plant Species *Ludwigia decurrens* Walter

**DOI:** 10.3390/plants10091973

**Published:** 2021-09-21

**Authors:** Denny Kurniadie, Ryan Widianto, Dedi Widayat, Uum Umiyati, Ceppy Nasahi, Hisashi Kato-Noguchi

**Affiliations:** 1 Department of Agronomy, Faculty of Agriculture, Universitas Padjadjaran, Jl. Raya, Bandung Sumedang Km 21, Jatinangor, Sumedang 45363, Jawa Barat, Indonesia; ryanwidianto21@hotmail.com (R.W.); widayatdedi@yahoo.com (D.W.); umiyati_uum@yahoo.com (U.U.); 2 Department of Plant Pest and Diseases, Faculty of Agriculture, Universitas Padjadjaran, Jl. Raya, Bandung Sumedang Km 21, Jatinangor, Sumedang 45363, Jawa Barat, Indonesia; ceppynasahi@yahoo.com; 3 Department of Applied Biological Science, Faculty of Agriculture, Kagawa University, Miki, Kagawa 761-0795, Japan

**Keywords:** herbicide resistance, invasive, *Ludwigia decurrens*, penoxsulam, rice weed

## Abstract

*Ludwigia decurrens* Walter is a dicotyledonous plant belonging to the family Onagraceae. It is native to Central Eastern USA but has been spreading quickly and has naturalized in aquatic and riparian ecosystems (including rice paddy fields) in many countries; therefore, it is now considered an invasive noxious weed. *L. decurrens* is highly competitive with rice and causes a significant reduction in rice production. The objective of the present study was to evaluate the efficacy of the herbicide penoxsulam for the control of *L. decurrens* in rice fields. The seeds of *L. decurrens* were collected from four villages in Indonesia, and penoxsulam was applied to *L. decurrens* in seven dosages (0, 2.5, 5, 10, 20, 40, and 80 g a.i. ha^−1^) 3 weeks after seed sowing. The plant populations from Hegarmanah, Jatisari, and Joho showed complete mortality at the recommended dosage of penoxsulam (10 g a.i. ha^−1^). However, the plants from Demakan grew, flowered, and produced seeds 56 days after treatment with 40 g a.i. ha^−1^ of penoxsulam. The resistance index value of the population was 36.06. This is the first report of a penoxsulam-resistant weed from a dicotyledonous plant species and also the first report of a herbicide-resistant population of *L. decurrens*. The appearance of herbicide-resistant *L. decurrens* is a serious issue from both an environmental and an economic perspective, especially since protected forest and freshwater ecosystems are located at a short distance from the study area. Further research is needed to evaluate herbicide mixtures and/or the rotation of herbicide action sites. The identification of the penoxsulam-resistance mechanism in *L. decurrens* is also necessary to develop a herbicide resistance management strategy.

## 1. Introduction

*Ludwigia decurrens* Walter, belonging to the Onagraceae family, is a synonym of *Jussiaea decurrens* Walter D.C. It is an annual or woody perennial herb that stands upright reaching 2 m of height, with alternate branches. The leaves are opposite, narrowly elliptical, 4–12 cm long, and 1–3 cm wide. A single flower with four bright yellow petals is produced in the upper leaf axis. A seed capsule develops immediately below the flower and contains up to 1000 seeds per capsule [1,2,3]. The species grows in wetlands including paddy fields, riverbanks, ponds, and slow-moving streams [1,4]. It has adapted to these aquatic habitats through the development of rhizomes with aerenchyma [5]. 

*L. decurrens* is native to Central Eastern USA but has been introduced accidentally into many countries in South and East Asia and Africa, where it is now considered an invasive noxious weed [1,2,4,6,7]. It produces a large number of seeds and spreads rapidly in the wet zone through seeds and plant fragments floating on water [1,4,8]. It has also allelopathic properties. The exudates of the plants inhibited the growth of *Corchorus olitorius* L. and increased its mortality [9]. Allelopathy may also be involved in *L. decurrens* invasion [10,11,12]. The severe invasion of some plant species often causes a significant reduction of biodiversity in the invaded ecosystems [13,14]. The risk of a negative environmental impact for *L. decurrens* is high because of its pest dispersal potential [4].

*L. decurrens* emerges along with rice seedlings and grows in rice paddy fields. It is highly competitive with rice due to its fast growth rate and a life cycle similar to that of rice and causes a significant reduction in rice production [1,15]. *L. decurrens* suppresses the development of tillers, panicles, leaves, and spikelets of rice plants. Consequently, the risk of an economic impact due to lower crop yields and quality is high [4]. For example, *L. decurrens* has been reported to reduce rice grain yield by around 30% [15]. Penoxsulam is one of the pre-emergence herbicides widely used in rice cultivation [16]. It is an acetolactate synthase (ALS; EC 4.6.3.8) inhibitor and is an efficient broad-spectrum herbicide against grass and broadleaf weeds [17]. This herbicide has been effective in controlling *L. decurrens*. However, excessive application of herbicides increases the potential to develop resistant weeds [18,19,20]. The appearance of herbicide-resistant weeds was predicted by Harper in 1956 [21], and a herbicide resistant-weed was first observed in sugarcane plantations in Hawaii [22]. Following this, triazine-resistant *Senecio vulgaris* L. [23] and 2,4-D-resistant *Convolvulus arvensis* L. [24] were recorded. Currently, 264 herbicide-resistant weed species—522 cases (species × site of action) for 164 herbicides—have been reported in 94 crops in 71 countries [25]. The present study was conducted due to reports of *L. decurrens* becoming difficult to control in rice paddy fields of Central Java, Indonesia. The efficiency of penoxsulam on *L. decurrens* collected from four Central Java villages was evaluated, and a penoxsulam-resistant *L. decurrens* population was confirmed for the first time.

## 2. Materials and Methods

### 2.1. Plant Materials 

Four locations (lowland rice cultivation areas) were selected for *L. decurrens* seed collection; (1) Jatisari village (Jatisari District, Karawang Regency, West Java Province; 6°22′41.8″ S, 107°31′42.1″ E), (2) Joho village (Mojolaban District, Sukoharjo Regency, Central Java Province; 7°35′25.2″ S, 110°53′39.7″ E), (3) Demakan village (Mojolaban District, Sukoharjo Regency, Central Java Province; 7°41′19.5″ S, 110°49′58.0″ E), and (4) Hegarmanah village (Jatinangor District, Sumedang Regency, West Java Province; 6°54′50.1″ S, 107°46′20.1″ E). At each location, seeds were collected from several plants in August 2020 and mixed well. The seeds were then cleaned and dried in the sun for one week to reduce the moisture and increase the maturity of the seeds.

### 2.2. Penoxsulam Dose–Response Experiment

Penoxsulam dose–response experiments were carried out using the whole-plant pot test dose–response method [26]. Pots (20 cm in diameter) were filled with paddy soil after sterilization using an autoclave at 120 °C and 15 Psi for 2 h so that only *L. decurrens* seeds were allowed to germinate and grow. Then, 10–20 seeds of *L. decurrens* were sowed on the soil surface in the pots. Water was applied regularly, and the water level was maintained at 0.5 cm above the soil surface to keep an adequate amount of water in the soil during the experiments. Three weeks after sowing, plants grew approximately 10 mm in height with two leaves, and penoxsulam (Clipper 25 OD, 25 g L^−1^, Dow Agro Science Indonesia) was applied at 7 dosages (0, 2.5, 5, 10, 20, 40, and 80 g a.i. ha^−1^) by using a semi-automatic knapsack sprayer with a flat fan nozzle at a pressure of 138 kPa. The recommended dosage of the herbicide is 10 g a.i. ha^−1^. After 28 days from herbicide application, *L. decurrens* was harvested, except for one plant per pot. Five plants among the harvested plants for each treatment were used for the determination of their dry weight. The remaining plant was grown for 56 days after herbicide application.

### 2.3. Statistical Analysis

The experimental design used in this experiment was a 2-factor split plot (main factor; populations, subfactor; penoxsulam dosage) with three replications. The percentage of weed damage was obtained from the comparison between the dry weight of herbicide-treated weeds (T) and the dry weight of control weeds, which were not treated with the herbicide (C), using the following equation (1) [27]:

Percentage of damage (%) = [1 − (T/C)] × 100(1)

The percentage of damage was analyzed by ANOVA. The interaction between weed populations and herbicide doses was analyzed based on a *p*-value < 0.05. If an interaction occurred, Tukey’s test was used to identify significant differences.

The penoxsulam dose required for 50% growth reduction (GR_50_) was obtained by nonlinear regression using the log-logistic dose response equation (2) as described in [28]:

Y= c + (d−c)/[1 + (X/GR_50_)^b^] (2)

where Y represents dry weight at herbicide dose (X), whereas c and d denote lower and upper limits, respectively, and b is the slope of the response curve. The dose–response analysis was performed using *OriginPro* 9.0. 

## 3. Results

**Figure 1:**
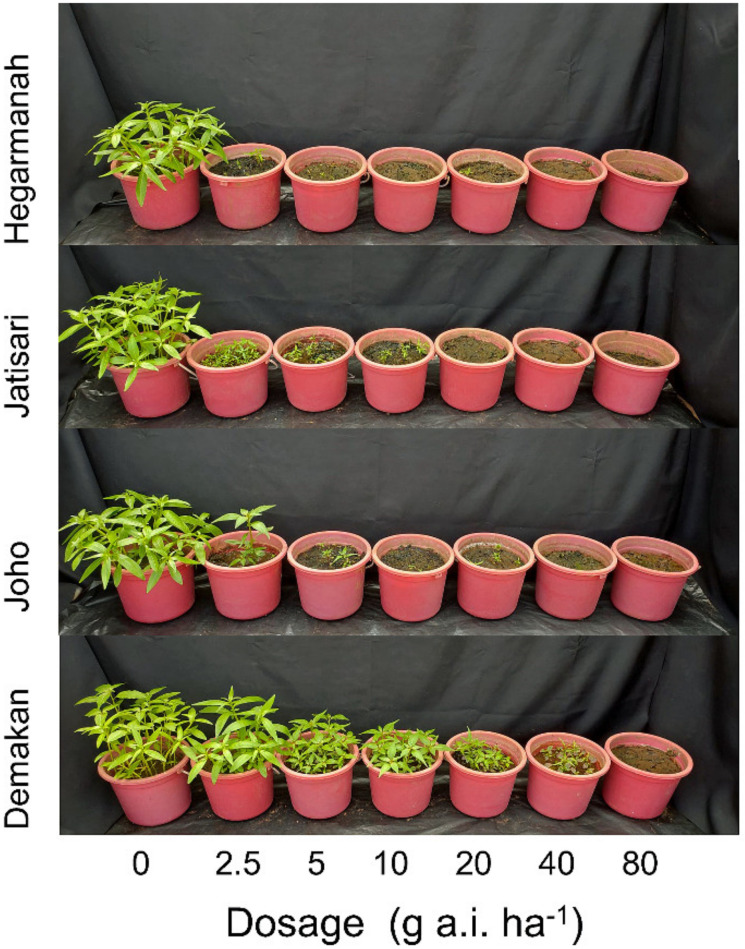
Effect of penoxsulam on four populations of *L. decurrens* 28 days after herbicide application.

**Figure 2:**
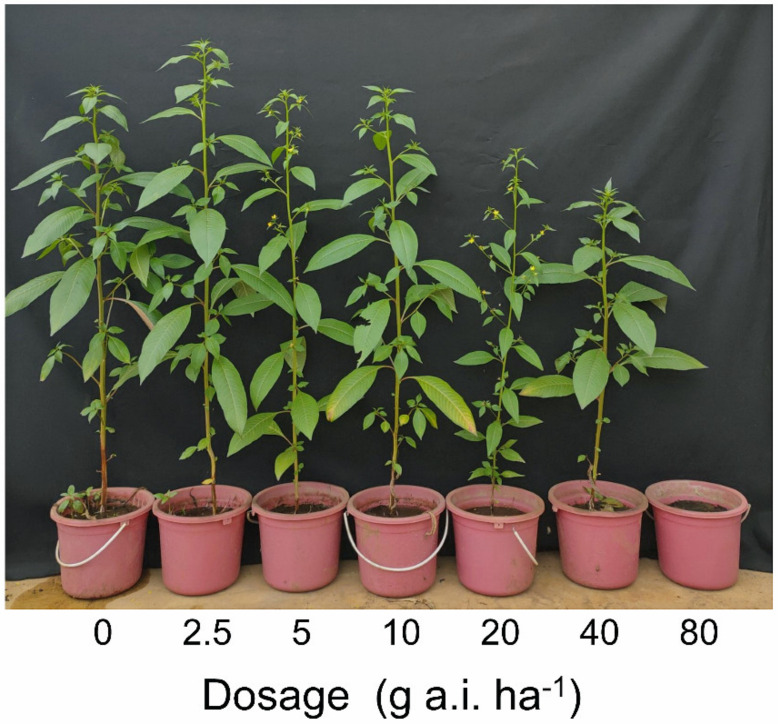
Effect of penoxsulam on the Demakan population of *L. decurrens* 56 days after herbicide application.

The recommended dosage of penoxsulam (10 g a.i. ha^−1^) controlled 100% of *L. decurrens* populations obtained from Hegarmanah, Jatisari, and Joho villages. However, the damage to the population from Demakan village was 38.24% and 72.85% at the recommended dosage and at the four-fold recommended dosage of 40 g a.i. ha^−1^, respectively (Table 1). The populations of Hegarmanah, Jatisari, and Joho showed complete mortality at the recommended dosage of penoxsulam (Figure 1). The plants of the Demakan population grew and produced flowers, and seeds were obtained 56 days after the treatment with penoxsulam (Figure 2).

**Figure 3:**
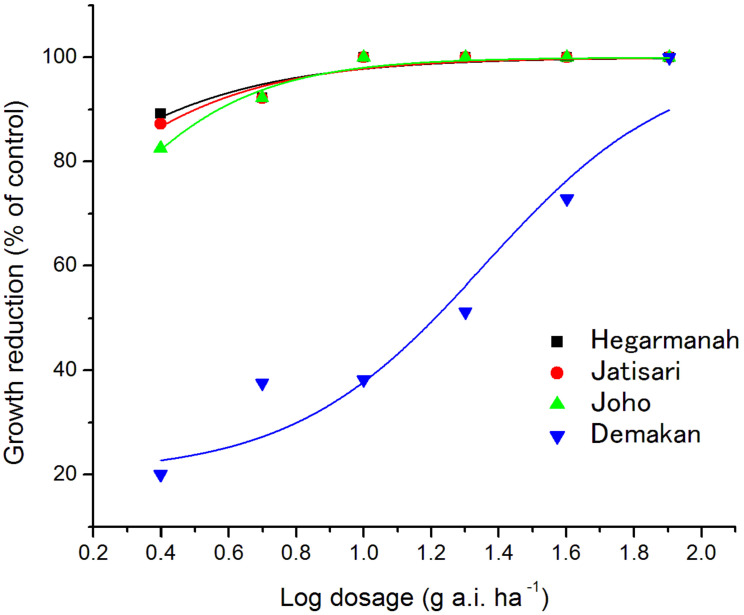
Growth reduction curves of four populations of *L. decurrens* against penoxsulam dose.

The GR_50_ value of penoxsulam for the Hegarmanah population was 0.63 g a.i. ha^−1^ (Figure 3, Table 2). As reported when interviewed, farmers of Hegarmanah village have never used any herbicides. Other farmers of Jatisari, Joho, and Demakan villages have applied penoxsulam twice per rice growing season (7 days and 21 days after planting), with three rice cultivations per year over the last 10 years. Therefore, the level of the resistance index (R/S) for penoxsulam was calculated by the ratio of the GR_50_ value of the Hegarmanah population (S; susceptible) to those of the other three populations (R; resistant), and the plants were classified as susceptible (R/S < 2), low-resistance (R/S = 2–6), moderate-resistance (R/S = 6–12), and high-resistance (R/S > 12) [29]. The R/S index of Demakan was 36.06, which indicates that the resistance of Demakan to penoxsulam was 36.06-fold greater than that of Hegarmanah (Table 2). Therefore, the efficiency of penoxsulam against the Demakan population was very low, and the population was classified as resistant, whereas the Jatisari and Joho populations were classified as susceptible because their R/S was less than 2.

## 4. Discussion

An invasive plant species, *L. decurrens*, has been spreading quickly and naturalized into aquatic and riparian ecosystems including rice paddy fields in many countries [1,2,4,6,7]. This species has been reported to occupy 50% of the invaded plant community, and thus is considered one of the most aggressive weed species [3,8]. Consequently, the risks associated with *L. decurrens* invasion are high from both an environmental and an economic perspective [4]. Management of *L. decurrens* relies on physical and chemical methods [30], and penoxsulam has effectively controlled *L. decurrens* so far. However, we identified penoxsulam-resistant *L. decurrens* plants from a Demakan population for the first time in this study (Figure 1, Table 1). The resistance index value of the population was 36.06 (Table 2), and the plants made flowers and produced seeds (Figure 2). 

The possibility of the herbicide-resistant *L. decurrens* to spread and dominate in the surrounding area may be high because of the properties of the species [3,8]. The total area of rice paddy fields is 20,460 ha in the Sukoharjo regency, which the Demakan village belongs to [31]. In addition, the protected forest Gunung Merbabu and the Gajah Mungkur water reservoir are located 20 and 50 km from the study area of the Demakan village, respectively. *L. decurrens* has been recorded to spread up to 120 km through seeds and plant fragments floating on water [32]. Therefore, the appearance of herbicide-resistant *L. decurrens* could be a possible threat for the nearby protected areas.

The excess application of herbicides with a single mode of action increases the risk of causing gene mutations leading to the appearance of herbicide-resistant weeds. Many other factors, such as cropping system, weed potentiality, and environment, also increase the appearance of resistant weeds [18,19,20]. *Echinochloa crus-galli* (L.) P.Beauv. is one of the most dangerous weeds in crop production and is mainly controlled by herbicides [25,33,34]. Continuous and excess applications of herbicides such as ALS-inhibiting and acetyl-CoA carboxylase (ACCase; E.C.6.4.1.2)-inhibiting herbicides have originated multiple-herbicide-resistant *E. crus-galli* populations [25,33,34,35,36,37]. 

ALS is the enzyme in the biosynthetic pathway of the branched-chain amino acids valine, leucine, and isoleucine [38]. ALS is an important target site for at least four structurally distinct classes of herbicides, i.e., sulfonylureas [39,40], imidazolinones [41], triazolopyrimidines [42], and pyrimidinylsalicylates [43]. The ALS inhibitor penoxsulam has become the most widely used herbicide in rice cultivation since its introduction [16,37]. There have been 103 cases of weed resistance to ALS herbicides in rice cultivation [18,25]. *Limnocharis flava* (L.) Buchenau from Malaysia’s Penang Island was found to be resistant to several ALS enzyme-inhibiting herbicides, such as the sulfonylurea and imidazolinone herbicide groups. *Monochoria vaginalis* (Burm.f.) Kunth from rice fields with an intensive use of herbicides in Chonnam, Korea, was resistant to the sulfonylurea and pyrimidinylsalicylate groups. Therefore, some ALS herbicide-resistant weed species showed resistance to multiple herbicides [18,25]. Based on the data from herbicide-resistant weed species, 7 penoxsulam resistant-weed species with 19 cases have been reported: *Echinochloa crus-galli*, *Echinochloa oryzicola* Vasing., *Cyperus iria* L., *Cyperus difformis* L., *Cyperus esculentus* L., *Fimbristylis miliacea* (L.) Vahl., and *Sagittaria montevidensis* Cham. & Schltdl. Of the 19 penoxsulam resistance cases, 17 experienced cross or multiple resistance [25]. All penoxsulam-resistant weed species reported belong to monocotyledonous plant species, and *Echinochloa crus-galli* was the most frequently reported. Ours is the first report of a penoxsulam-resistant weed from dicotyledonous plant species and also the first report of a herbicide-resistant population of *L. decurrens* [25]. 

According to Heap [18,25], there are five primary mechanisms of herbicide resistance: (1) target-site resistance, (2) enhanced metabolism of herbicides, (3) decreased absorption and translocation of herbicides, (4) sequestration of herbicides, and (5) gene amplification of target genes. Resistance to ALS inhibitors, ACCase inhibitors, triazine and dinitroaniline herbicides is often caused by target-site resistance mechanism. Target-site resistance may be caused by gene mutations that modify the herbicide binding site, inhibiting herbicide binding [18,37]. Mutations in ALS inhibitors have been reported in the amino acids Thr_102_ (four cases), Ala_103_ (one case), and Pro_103_ (21 cases) of the ALS protein (the amino acid number is standardized according to the sequence of the ALS protein in *Arabidopsis thaliana* (L.) Heynh.) in various weed species [25]. The high frequency of herbicide application can increase the possibility of gene mutations that modify the herbicide binding sites [18,37]. As describe earlier, farmers of Demakan villages applied penoxsulam twice during each rice growing season with three rice cultivations per year for over 10 years, without herbicide rotation. Therefore, it is possible that penoxsulam resistance in *L. decurrens* may have been caused by mutations leading to the modification of the herbicide binding sites, causing target-site resistance.

The appearance of resistance to multiple herbicides in weeds is a serious issue for weed control. In fact, populations of *Lolium rigidum* Gaud. were found to be resistant in seven herbicide action sites [18,25,44]. Populations of *Amaranthus palmeri* S.Wats. showed resistance to PSII inhibitors, ALS inhibitors, ACCase inhibitors, 4-HPPD inhibitors, glyphosate, and others [18,25]. Population of *Ludwigia prostrata* Roxb., which belongs to the same genus of *L. decurrens,* from South Korea, showed multiple resistance to ALS inhibitor herbicides, such as sulfonylureas, imidazolinones, and pyrimidinylsalicylates [18,25]. Therefore, further investigation of resistance to multiple herbicides in *L. decurrens* is necessary.

The most common herbicide resistance management strategy may be to rotate herbicide action sites [45,46]. This practice may delay the evolution of herbicide resistance. The employment of herbicide mixtures, such as combinations of ALS inhibitors, ACCase inhibitors, glyphosate, glufosinate, 4-HPPD inhibitors, and synthetic auxins may also delay the appearance of resistance. However, it is essential to identify the penoxsulam resistance mechanism in *L. decurrens* to design an efficient herbicide resistance management strategy.

## 5. Conclusions

*L. decurrens,* originated from Central Eastern USA, has naturalized in many countries as a noxious invasive weed species in aquatic and riparian ecosystems including rice paddy fields. The ALS inhibitor herbicide penoxsulam has effectively controlled *L. decurrens* so far. However, a penoxsulam-resistant population was identified in Central Java, Indonesia, which is close to protected areas. Its resistance index value was 36.06. These resistant plants grew, flowered, and produced seeds 56 days after treatment with 40 g a.i. ha^−1^ of penoxsulam, a dose four-fold greater than the recommended one. The farmers in the area applied penoxsulam twice per rice growing season with three rice cultivations per year over the last 10 years, without herbicide rotation. Therefore, it is possible that the penoxsulam-resistant *L. decurrens* emerged as a consequence of the modification of the herbicide binding sites in the enzyme. The employment of herbicide mixtures and/or the rotation of herbicide action sites should be considered. The identification of the penoxsulam-resistant mechanism in *L. decurrens* is also necessary for an effective herbicide resistance management strategy.

## Figures and Tables

**Table 1:** Effect of penoxsulam on the percentage of damage to *L. decurrens*.

	Penoxsulam Dosage (g a.i. ha^−1^)						
Population	0	2.5	5	10	10	40	80
Hegarmanah	0 a,A	89.18 a,B	92.22 a,B	100 a,B	100 a,B	100 a,B	100 a,B
Jatisari	0 a,A	87.22 a,B	92.17 a,B	100 a,B	100 a,B	100 a,B	100 a,B
Joho	0 a,A	82.53 b,B	92.23 a,C	100 a,C	100 a,C	100 a,C	100 a,C
Demakan	0 a,A	20.11 c,B	37.57 b,C	38.24 b,C	51.2 b,D	72.85 b,E	100 a,F

The percentage of damage was calculated from the comparison between the dry weight of herbicide-treated *L. decurrens* and that of control *L. decurrens*. Damage of 100% indicates complete inhibition. Values in each column followed by the same lowercase letters (vertical direction) and uppercase letters (horizontal direction) are not significantly different at *p* < 0.05 according to the Tukey test.

**Table 2:** Herbicide dose required for 50% reduction (GR_50_) of dry biomass and resistance index.

Population	c	d	b	r^2^	GR_50_ (g a.i. ha^−1^)	Resistance Index	Level of Resistance
Hegarmanah	20.11	100	1.29	0.86	0.63	-	-
Jatisari	20.11	100	1.42	0.91	0.81	1.28	Susceptible
Joho	20.11	100	1.75	0.97	1.23	1.95	Susceptible
Demakan	20.11	100	1.53	0.92	22.72	36.06	Resistance

GR_50_ values of penoxsulam were obtained by nonlinear regression using the log-logistic dose–response equation. The resistance index against penoxsulam was calculated by the ratio of the GR_50_ value of the Hegarmanah population to those of the other three populations.

## Data Availability

No supporting data in this study.
